# Post-exposure prophylaxis with SA58 (anti-SARS-COV-2 monoclonal antibody) nasal spray for the prevention of symptomatic COVID-19 in healthy adult workers: a randomized, single-blind, placebo-controlled clinical study*

**DOI:** 10.1080/22221751.2023.2212806

**Published:** 2023-05-25

**Authors:** Rui Song, Gang Zeng, Jianxing Yu, Xing Meng, Xiaoyou Chen, Jing Li, Xiaoliang Xie, Xiaojuan Lian, Zhiyun Zhang, Yunlong Cao, Weidong Yin, Ronghua Jin

**Affiliations:** a Beijing Ditan Hospital, Capital Medical University, Beijing, People’s Republic of China; b Sinovac Biotech Co., Ltd., Beijing, People’s Republic of China; c Sinovac Life Sciences Co., Ltd., Beijing, People’s Republic of China; d Biomedical Pioneering Innovation Center (BIOPIC), Peking University, Beijing, People’s Republic of China; e Changping Laboratory, Beijing, People’s Republic of China

**Keywords:** Monoclonal antibodies, Post-exposure prophylaxis, SARS-CoV-2, COVID-19, Clinical trial, China

## Abstract

Monoclonal antibodies (mAbs) and the post-exposure prophylaxis (PEP) with mAbs represent a very important public health strategy against coronavirus disease 2019 (COVID-19). This study has assessed a new Anti-SARS-COV-2 mAb (SA58) Nasal Spray for PEP against COVID-19 in healthy adults aged 18 years and older within three days of exposure to a SARS-CoV-2 infected individual. Recruited participants were randomized in a ratio of 3:1 to receive SA58 or placebo. Primary endpoints were laboratory-confirmed symptomatic COVID-19 within the study period. A total of 1222 participants were randomized and dosed (SA58, *n* = 901; placebo, *n* = 321). Median of follow-up was 2.25 and 2.79 days for SA58 and placebo, respectively. Adverse events occurred in 221 of 901 (25%) and 72 of 321 (22%) participants with SA58 and placebo, respectively. All adverse events were mild in severity. Laboratory-confirmed symptomatic COVID-19 developed in 7 of 824 participants (0.22 per 100 person-days) in the SA58 group vs. 14 of 299 (1.17 per 100 person-days) in the placebo group, resulting in an estimated efficacy of 80.82% (95%CI 52.41%−92.27%). There were 32 SARS-CoV-2 reverse transcriptase polymerase chain reaction (RT–PCR) positives (1.04 per 100 person-days) in the SA58 group vs. 32 (2.80 per 100 person-days) in the placebo group, resulting in an estimated efficacy of 61.83% (95%CI 37.50%−76.69%). A total of 21 RT–PCR positive samples were sequenced and all were the Omicron variant BF.7. In conclusion, SA58 Nasal Spray showed favourable efficacy and safety in preventing symptomatic COVID-19 or SARS-CoV-2 infection in adults who had exposure to SARS-CoV-2 within 72 h.

## Introduction

Postexposure prophylaxis (PEP) is the administration of chemicals or immunotherapeutic agents to prevent the development of infection or to slow the illness progression prior to the illness onset. PEP has been routinely recommended for several viral infections, including influenza virus [1], rabies virus [2], human immunodeficiency virus [3], hepatitis B virus[4] and varicella-zoster virus [5], especially for those who have higher risks of severe outcomes or mortality following infection [6].

Coronavirus disease 2019 (COVID-19) is a contagious condition caused by severe acute respiratory syndrome coronavirus 2 (SARS-CoV-2) that firstly emerged in December 2019 [7]. The on-going COVID-19 pandemic has led to high morbidity and mortality worldwide [8], resulting in 648 million laboratory-confirmed cases and 6.64 million deaths globally as of December 15, 2022 [9]. This substantial impact of COVID-19 has reshaped the world and profoundly changed public health practices, including the development of various prevention (e.g. vaccines) and therapeutic measures. Several passive immunotherapeutic antibodies against SARS-CoV-2 have since been generated, tested, and moved into clinical trials[10]. However, due to the high frequency of mutations, newly emerged SARS-CoV-2 variants have been circulating in the population (e.g. Omicron variants). These variants have developed significant escape properties, resulting in several monoclonal antibodies (mAbs) that had initially been authorized to treat COVID-19 or used as prophylaxis, to be discontinued (i.e. *Bamlanivimab and Etesevimab, Evusheld, Bebtelovimab, Sotrovimab*, and *REGEN-COV*) [11,12]. As of 15 March 2023, no mAbs were approved as treatment or PEP against COVID-19 in the US. As broad-spectrum mAbs and PEP represent a very important public health strategy against COVID-19 outbreak, especially among high-risk populations who are vulnerable to severe disease following infection, it is important to develop and evaluate more potential broad-spectrum mAbs, which can be protective against the SARS-CoV-2 Omicron sub-lineages circulating in the population and other upcoming variants.

SA58 Nasal Spray, a broad-spectrum anti-SARS-CoV-2 mAb, was developed by Sinovac Life Sciences Co., Ltd. This antibody was identified from a large collection of broad *sarbecovirus* mAbs isolated from SARS-CoV-2-vaccinated SARS convalescents. It is highly resistant to mutations causing immune evasion of several earlier discontinued mAbs, and has been shown to potently neutralize ACE2-utilizing *sarbecoviruses*, including circulating Omicron variants (i.e. BA.1, BA.2, BA.2.12.1, BA.3, BA.4/BA.5, BF.7, and BQ.1.1) in in vitro neutralizing and in animal challenge studies [13,14]. Though the neutralization efficacy of SA58 against the most recent emerging variants (i.e. XBB and BA.2.75 sublineages) was shown to be reduced in in vitro neutralizing study [14], it does not exclude it as a potent mAb. The early pharmacokinetic results in human volunteers showed that SA58 was safe, and that the half-life of SA58 administered intranasally was 2–4 h in the nasal cavity and 12–27 h in the nasopharynx, with no detectable drug components in the blood (below the detection limit of the method used). In previous study on the high-risk population of medical workers [15], the most common encountered symptoms were runny nose, nasal mucosal dryness, nasal congestion, and headache post administration, suggesting good safety and tolerability of a SA58 Nasal Spray. More information about the effect of SA58 Nasal Spray administered as PEP against symptomatic COVID-19 or SARS-CoV-2 infection was needed. Therefore, the aim of this study was to estimate the efficacy of SA58 Nasal Spray in preventing symptomatic COVID-19 in healthy adults who had exposure to individuals with laboratory-confirmed SARS-CoV-2 infection within 72 h.

## Materials and methods

### Study design and participants

This randomized, single-blind, placebo-controlled clinical trial evaluated the efficacy and safety of the SA58 Nasal Spray in healthy adult workers within 72 h of contact with a SARS-CoV-2-infected individual in Beijing, China. The study was conducted from 26 November 2022 to 9 December 2022 at 21 construction sites (median number of workers = 45, range = 9–235) that had COVID-19 outbreaks reported within two days of the first COVID-19 case notified. The construction sites setting was selected because we wanted to evaluate SA58 firstly in healthy adults before expanding to other high-risk populations, and we could enrol a large number of healthy adult workers on construction sites during a short period of time. Moreover, prior to the study the Chinese government sticked on strict containment measures against COVID-19 before issuing the new public health policy on 7 December 2022 [16], and there were several outbreaks in construction sites notified to Beijing health authority. Since most COVID-19 outbreaks firstly appeared in institutions, like construction sites, factories, hospitals and other closed space settings (i.e. markets and nursing home) before spreading into communities, early and timely control of COVID-19 outbreaks in institutions is thus of greater significance to government. All participants in this study were volunteers and provided written informed consent before enrollment. The clinical trial protocol and informed consent form were approved by the Ethics Committee of Beijing Ditan Hospital, Capital Medical University (reference no., DTEC-YW2022-024-01). The study was registered with ClinicalTrials.gov (NCT05667714).

In this study, all healthy workers in the 21 construction sites were offered the opportunity to participate based on the following inclusion/exclusion criteria. Participants were eligible for inclusion if they were aged 18 years or older, had potential exposure to a well-identified individual with laboratory-confirmed SARS-CoV-2 infection (index case), and if the presumable contact occurred within 72 h of the positive reverse transcriptase polymerase chain reaction (RT–PCR) test of the index case. The key exclusion criteria were individuals with known history of severe allergies or reaction to any component of inhaled SA58 Nasal Spray; those currently pregnant, lactating, or expected to be pregnant during the study period; those who participated in any kind of clinical trials of SARS-CoV-2 neutralizing antibody injections in the preceding 180 days before screening or participated in any investigational medicinal product in the preceding four weeks before screening; were unable to take nasal spray inhalation; had reported fever at enrollment or axillary temperature of more than 37.0°C; had severe neurological disease (e.g. epilepsy, convulsions, or seizures) or psychosis, or family history of psychosis; or had any other significant chronic disease, disorder, or finding that, in the judgement of the investigator, significantly increased the risk to the participant because of participation in the study, affected the ability of the participant to participate in the study, or impaired interpretation of the study data. Full eligibility criteria are provided in the Supplementary Material. A nasopharyngeal swab, nasal swab, or throat swab was collected at baseline for detection of SARS-CoV-2 nucleic acid by RT–PCR tests, but the results of baseline RT–PCR tests were not used to determine the eligibility of participants.

### Outcomes

The primary efficacy endpoint was the laboratory-confirmed symptomatic COVID-19 that occurred during the case monitoring period between 24 h after the first administration and 24 h after the last administration of SA58 or placebo. COVID-19 case was defined based on symptoms (see Supplementary Table 1). Severe COVID-19 was defined based on the Protocol for Prevention and Control of COVID-19 (9th edition) issued by the National Health Commission of the People’s Republic of China, and we arbitrarily defined severe COVID-19 as severe case and very-severe case combined (see Supplementary Table 2) [17]. The safety endpoints included incidence of adverse events (AEs), serious AEs (SAEs), and AEs of special interest (AESIs).

### The investigational drug

The investigational drug SA58 was prepared into 2 ml prefilled sprayer (20 sprays per bottle), containing 5 mg of anti-SARS-CoV-2 mAb per ml. Placebo, only without anti-SARS-CoV-2 mAb as compared with SA58 Nasal Spray, was also prefilled into bottles with identical package that could not be easily distinguished by their appearance. The drug and placebo were self-administered by nasal sprays with a video instruction. When used, insert the sprayer nozzle into each nose nostril and press the pump to spray 0.1 ml of the nebulized solution into the nasal cavity. Each administration of the drug consisted of two sprays with one spray in each nostril, and a total of 1 mg antibody was administered into both nostrils. For an ordinary exposure day, 5–6 administrations of SA58 or placebo were recommended at an interval of 3–4 h per administration, with the last administration given before going to bed.

### Procedures

#### Randomization and masking

After enrollment, participants were randomized in a ratio of 3:1 to receive SA58 or placebo at the study site. A statistician (independent of the study) assigned treatment at random using a standard computer pseudorandom number generator. Given that our study was conducted in a COVID-19 outbreak setting, only the participant was blinded and unaware of what had been allocated. The sponsor/investigators were unblinded for treatment to meet the sites’ requirements of taking high standard of efforts to secure the safety of participants without any delay.

#### Administration of drugs

After confirmation of participants’ contact to a laboratory-confirmed SARS-CoV-2 infection, participants were classified into two groups according to their type of exposure to SARS-CoV-2, e.g. Group A participants with continuous exposure to COVID-19 in which potential contact to SARS-CoV-2 infected individual was not blocked by removing or eliminating the source of infection in the site, and Group B participants with one-time exposure in which contact to SARS-CoV-2 infected individual was immediately blocked by managing study participants in isolation facilities for highly infectious diseases.

For the purpose of PEP against continuous exposure to COVID-19, Group A participants were administered SA58 or placebo on every exposure day according to the recommended administration schedule and stopped administration three days after the source of infection had been eliminated or removed from the study site (e.g. all SARS-CoV-2 infected individual in the site had no symptoms and had negative RT–PCR testing results of SARS-CoV-2 for the last two consecutive days); for the purpose of PEP against one-time exposure in which the contact to the SARS-CoV-2 infected individual was immediately blocked, each participant was administered SA58 or placebo for a maximum of three days according to the recommended administration schedule.

#### Follow-up of adverse events

Participants self-monitored for safety post-administration and spontaneously reported adverse events (e.g. runny nose, sneezing, nasal congestion, nasal dryness, fever, headache, and fatigue) post-administration to study follow-up personnel. Predefined symptoms (solicited events) and other unspecified symptoms (unsolicited events) reported by the participants during the study period were recorded and verified at regular visits by the study investigators. Any SAEs and AESIs (e.g. allergic reaction, autoimmune reaction, and nasal and throat AEs of Grade two and above) were reported up to 30 days since enrollment. Adverse events were graded according to the US National Cancer Institute’s Common Terminology Criteria for Adverse Events (version 5.0) [18].

#### Monitoring of COVID-19 cases

To specifically measure the incidence of laboratory-confirmed symptomatic COVID-19 post-administration, study sites contacted participants daily to collect nasopharyngeal/throat/nasal swab and monitor information on symptoms of COVID-19. The case monitoring period extended from 24 h after the first administration to 24 h after the last administration of SA58 or placebo, during which participants were observed closely. If a participant had positive RT–PCR testing result, he/she was defined a SARS-CoV-2 RT–PCR positive participant (e.g. laboratory-confirmed SARS-CoV-2 infection). And if a SARS-CoV-2 RT–PCR positive participant had symptoms of COVID-19 (Supplementary Table 1), this was defined as a laboratory-confirmed symptomatic COVID-19 case. Both laboratory-confirmed SARS-CoV-2 infections and symptomatic COVID-19 cases were followed up to disease resolution, or up to negative RT–PCR tests on two consecutive days.

#### Laboratory methods

The nasopharyngeal/throat/nasal swabs were transferred at 2–8°C to the central laboratory and tested within 24 h of collection. We used real-time RT–PCR for detecting of SARS-CoV-2 in nasopharyngeal/throat/nasal swabs as per national guidelines [17]. The RT–PCR positive samples that had cycle threshold values <30 were sequenced to identify SARS-CoV-2 variants by using the methods provided in the supplementary material.

### Statistical analysis

We assumed a 5% secondary attack rate of symptomatic COVID-19 cases in the placebo group during our study period, and a true efficacy of 70% (equating to an attack rate of 1.5% with SA58). Allowing for a dropout rate of 10%, we calculated that a study population of approximately 2300 participants randomized in a 3:1 ratio to treatment or placebo would be sufficient to provide approximately 80% power to demonstrate the lower bound of the two-sided 95% confidence interval (CI) for efficacy to be >0.3.

The incidence rates of symptomatic COVID-19 per 100 person-days were calculated for the SA58 and placebo groups during the case monitoring period, by dividing the number of events with the total number of follow-up days. Crude incidence rate ratios (IRRs) comparing the SA58 and placebo groups were calculated. For the adjusted analysis, a Poisson regression model was used, with robust variance using the log of the follow-up days as an offset to estimate IRR of symptomatic COVID-19 cases with SA58 vs. placebo. Efficacy of treatment was calculated as 1 minus IRR and was presented with a two-sided 95% confidence interval (95%CI). In the final efficacy analysis, we excluded participants who tested positive for SARS-CoV-2 within 24 h by RT–PCR at the visit on Day one to account for people with early and undetectable infection when SA58 was administered and for an expected time lag between administration and RT–PCR screening assay. Kaplan–Meier curves were also presented for SA58 and placebo groups, with hazard ratios (HR) calculated by using Cox proportional hazard models. We also calculated the efficacy of SA58 against other outcomes, e.g. SARS-CoV-2 infection (SARS-CoV-2 RT–PCR positive during follow-up) and severe COVID-19 following the same methodology as for the primary efficacy end point. The robustness of our results was tested by performing separate analyses of efficacy among participants with different duration of follow-up days (namely <5 days vs. ≥5 days), by including participants who tested positive for SARS-CoV-2 by RT–PCR within 24 h of the first drug administration, and by restricting efficacy analyses to participants who had continuous exposure to COVID-19. For the comparison of individual-level variables at baseline, we used Student’s *t*-test or Wilcoxon test for continuous variables and the Chi-square test or Fisher’s exact test for categorical variables as appropriate. Adverse events were summarized descriptively as frequencies and percentages by type of event and severity. A two-sided *P*-value of <0.05 was considered statistically significant. We conducted all analyses in SAS (version 9.1.3).

## Results

### Characteristics of participants

**Figure 1.**
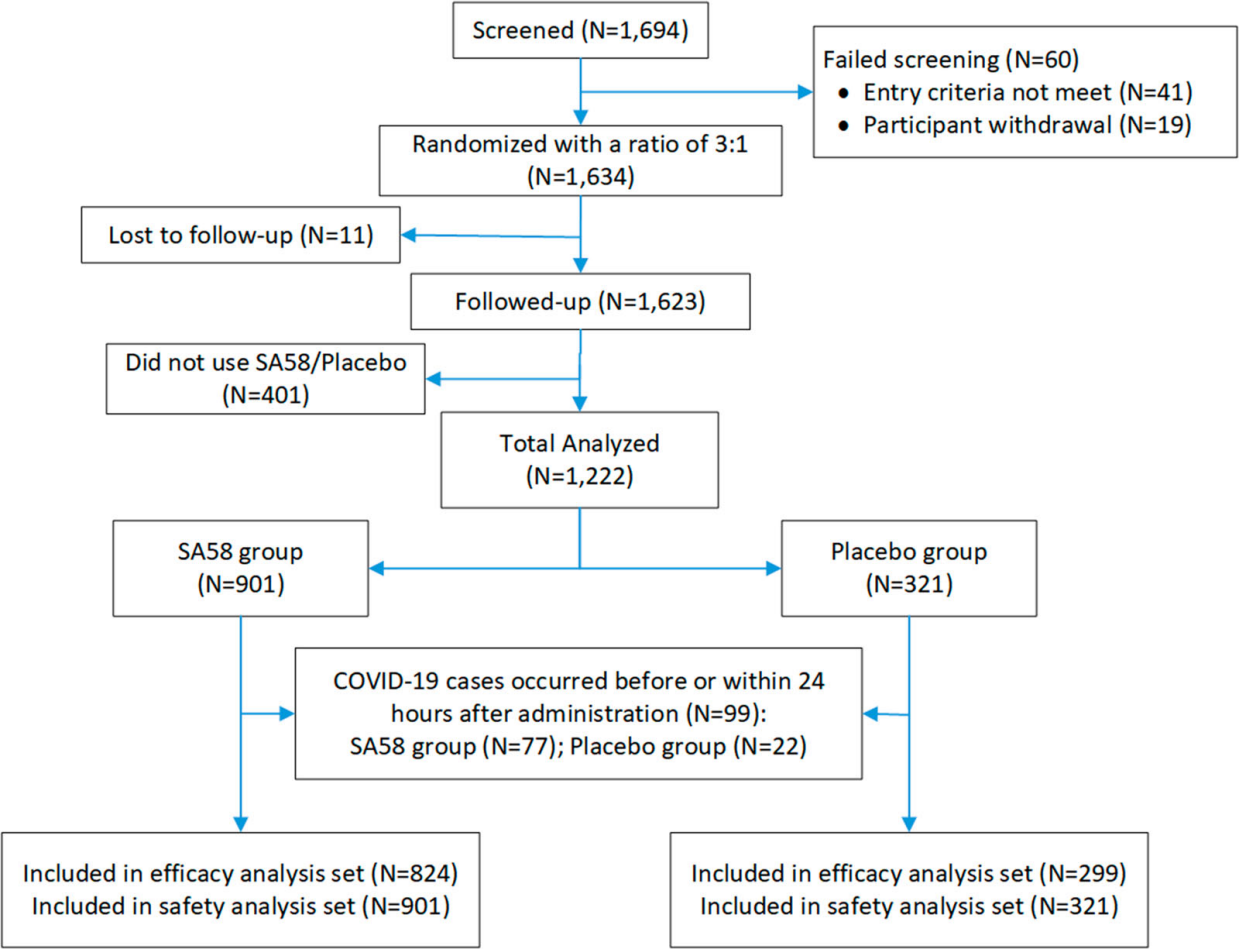
Participant disposition.

Because of the reopening of China and the lifting of the COVID-19 restrictions on 7 December 2022, the study participants left study sites and our study ended early before our target sample size was met. In total, 1694 participants at 21 construction sites were screened and were confirmed to contact with a SARS-CoV-2 infected individual within 72 h of RT–PCR positive results of an index case. Of which, 60 were ineligible or withdrew early from study before study drug administration, and 1634 were randomized in a ratio of 3:1 to receive SA58 or placebo. After excluding 401 participants not administrated and 11 participants lost to follow-up, 1222 participants entered the full analysis set, with 901 participants in SA58 group and 321 in placebo group (Figure 1). The median age of the 412 persons who dropped out of the study was a little older (46.0 vs. 48.5, *p* < 0.001) than those who remained in the final analyses (Supplementary Table 3). The major reason for dropout were participants’ complacency about their health, with over two thirds believing that they would not be infected or seriously infected while the rest gave no reasons. No subject dropped out because of intolerance to the investigational drug.

In the full analysis set, the median age was 46 years (interquartile range, IQR = 35–52 years) in SA58 group and 46 years (IQR = 35–52 years) in placebo group, *p* = 0.9934. The majority of participants were males (*n* = 1090, 89%) and adults aged 18–59 years (*n* = 1197, 98%). A total of 6063 respiratory samples were collected (4463 in SA58 and 1601 in placebo group) and all of them were collected as throat swabs. At baseline, 39 (4%) of 901 participants in the SA58 group and 15 (5%) of 321 participants in the placebo group were SARS-CoV-2 RT–PCR positive, and no significant difference of SARS-CoV-2 RT–PCR positivity was identified between the two comparison groups before drug administration. There were 45 (4%) participants testing positive for SARS-CoV-2 by RT–PCR within 24 h of the first drug administration (38 in the SA58 and 7 in the placebo group). We excluded from the efficacy analysis these participants who tested positive before and within 24 h of the first drug administration (*n* = 99). Median duration of follow-up for participants was 2.25 days (IQR = 1.00–5.63 days) and 2.79 days (IQR = 1.33–6.00 days) for SA58 and placebo recipients respectively, *p* = 0.1869 (Table 1).

**Table 1.** Baseline characteristics of study participants (full analysis set).

Characteristics	SA58 (*n* = 901)	Placebo (*n* = 321)	*p*-value
Age, median (IQR), yr	46.0 (35-52)	46.0 (35-52)	0.9934
Age groups, yr, No. (%)			0.4718
18–59	881(98)	316(98)	
60+	20(2)	5(2)	
Female sex, No. (%)	100(11)	32(10)	0.5755
SARS-CoV-2 RT-PCR status, No. (%)			
Positive before administration	39(4)	15(5)	0.7544
Positive within 24 h of administration	38(4)	7(2)	0.1195
Duration of follow-up days, median (IQR)	2.25 (1.00-5.63)	2.79 (1.33-6.00)	0.1869

Note: IQR: interquartile range.

### Treatment efficacy

**Figure 2.**
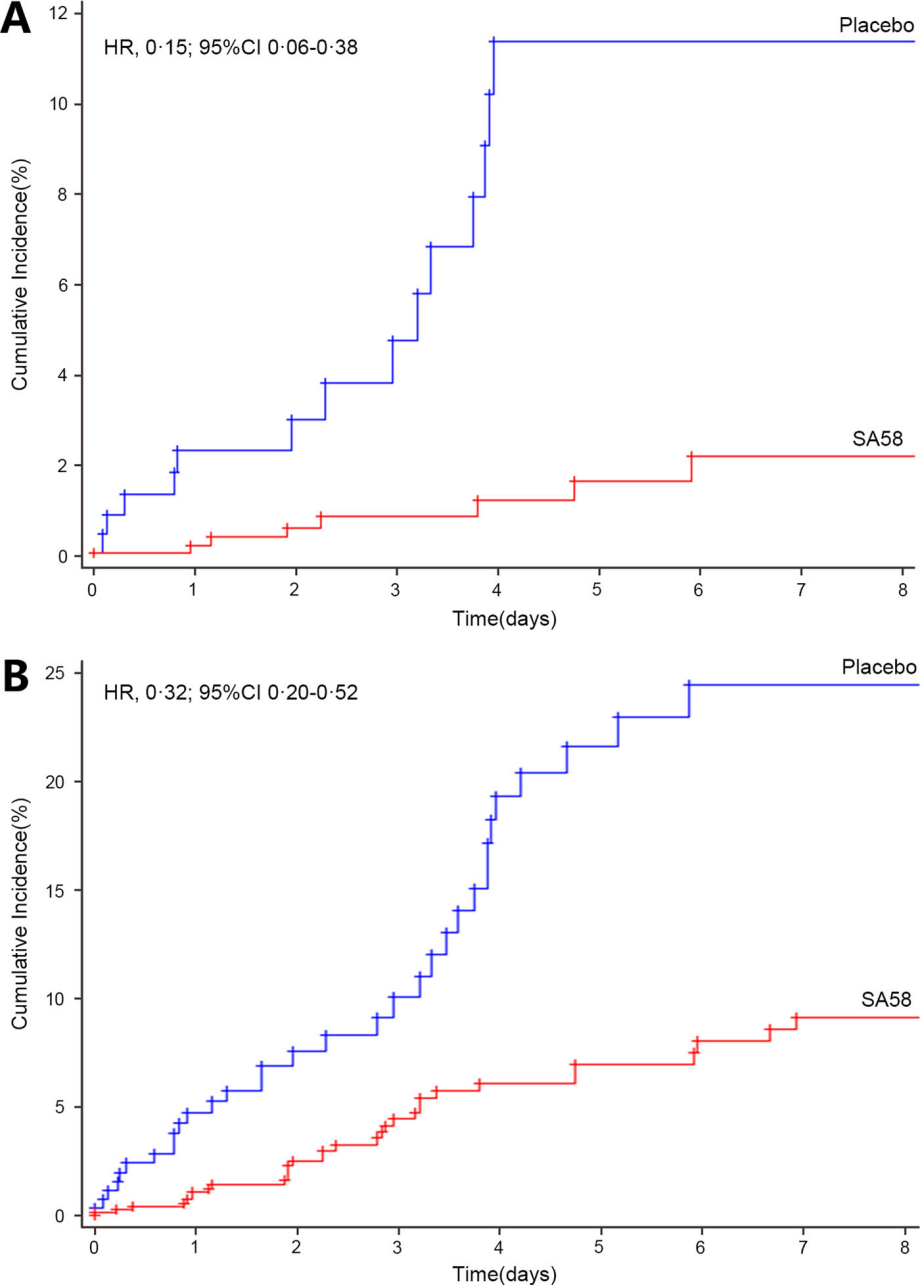
Time to first post-administration symptomatic COVID-19 (i.e. the Kaplan–Meier estimates of the cumulative risk of having COVID-19). Panel A. laboratory-confirmed symptomatic COVID-19; Panel B. SARS-CoV-2 RT-PCR positive. Abbreviation, HR, Hazard ratio; 95%CI, 95% confidence interval.

In the efficacy analysis, 1123 participants were followed for a total of 4362 days. The primary outcome laboratory-confirmed symptomatic COVID-19 developed in seven of 824 participants (incidence rate of 0.22 per 100 person-days) in the SA58-treated participants vs. 14 of 299 (incidence rate of 1.17 per 100 person-days) in the placebo group. The occurrence rate of symptomatic COVID-19 was significantly lower for SA58-treated participants vs. placebo, with a crude IRR of 0.19 (95%CI 0.08–0.48) and an estimated treatment efficacy of 80.82% (95%CI 52.41%−92.27%) (Table 2). Time to first post-administration symptomatic COVID-19 was shown in Figure 2(A). The majority of COVID-19 cases developed their symptoms within the first five days after enrollment (the median incubation period for COVID-19). The protective effectiveness of SA58 against SARS-CoV-2 infection was also explored by comparing the incidence of SARS-CoV-2 RT–PCR positive between SA58-treated participants vs. placebo. There were 32 SARS-CoV-2 RT–PCR positives (incidence rate of 1.04 per 100 person-days) in the SA58-treated participants vs. 32 (incidence rate of 2.80 per 100 person-days) in the placebo group, resulting in a crude IRR of 0.38 (95%CI 0.23-0.62) and an estimated efficacy of SA58 against SARS-CoV-2 infection of 61.83% (95%CI 37.50%−76.69%) (Table 2). The same patterns for time occurrence and SARS-CoV-2 RT–PCR positivity was observed for SA58- and placebo-treated participants as for symptomatic COVID-19 cases (Figure 2(B)). A total of 21 RT–PCR positive samples were sequenced. 21 lineages of SARS-CoV-2 variants were identified, and all were the Omicron variant BF.7 (Supplementary Figure 1). No severe COVID-19 or death developed in the study participants during case monitoring period.

**Table 2.** Occurrence of SARS-CoV-2 RT-PCR positive and laboratory-confirmed symptomatic COVID-19 in SA58- and placebo-treated participants.

Variable	SA58 (*n* = 824)			Placebo (*n* = 299)			Protective efficacy of treatment (95% CI)
	No. of events	Person-days at risk	Incidence rate (per 100 person-days)	No. of events	Person-days at risk	Incidence rate (per 100 person-days)	
SARS-CoV-2 RT-PCR positive	32	3078	1.04	32	1144	2.80	61.83% (37.50%−76.69%)
Symptomatic COVID-19	7	3162	0.22	14	1200	1.17	80.82% (52.41%−92.27%)
Severe COVID-19	0	3162	0	0	1200	0	NA

Sensitivity analyses showed similar efficacy results by performing separate analyses in participants with a follow-up duration of <5 days (Supplementary Table 4), participants with a follow-up duration of ≥5 days (Supplementary Table 5), and participants who had continuous exposure to COVID-19 (Supplementary Table 6), though there was significant drop of efficacy when including participants who tested positive for SARS-CoV-2 by RT–PCR within 24 h of the first drug administration, from 80.82% to 50.30% for Symptomatic COVID-19, and from 61.83% to 32.69% for SARS-CoV-2 infection (Supplementary Table 7).

### Adverse events

In the safety analysis, ≥1 AE was reported by 221 of 901 (25%) and 72 of 321 (22%) participants in the SA58 and placebo groups, respectively. All of the reported AEs were Grade one events as judged by study investigator. No SAEs and AESIs were reported by study participants, and no AEs led to study withdrawal or death during the short monitoring period. The most common AEs included nasal mucosal dryness (5%), runny nose (3%), fever (3%), nasal congestion (3%), headache (2%), cough (2%), and throat dryness (2%) etc. There was no significant difference in the occurrence of the above AEs between the SA58 and placebo groups (Table 3).

**Table 3.** Summary of adverse events.

Characteristics	SA58 (*n* = 901)	Placebo (*n* = 321)	*p*-Value
Treatment Emergent Adverse Events (TEAE)	221 (25)	72 (22)	0.4934
Nasal mucosal dryness	50 (6)	15 (5)	0.6640
Runny nose	29 (3)	11 (3)	0.8556
Headache	26 (3)	4 (1)	0.1398
Fever	22 (2)	11 (3)	0.4216
Nasal congestion	21 (2)	10 (3)	0.4148
Sneezing	20 (2)	5 (2)	0.6465
Cough	20 (2)	9 (3)	0.5277
Throat dryness	20 (2)	8 (2)	0.8283
Dizziness	18 (2)	5 (2)	0.8117
Sore throat	17 (2)	4 (1)	0.3133
SAEs	0	0	1
AESI	0	0	1

Note: AEs: adverse events; SAEs: severe adverse events; AESI: adverse events of special interest (i.e. allergic reaction, autoimmune reaction, and nasal and throat AEs of Grade 2 and above).

Adverse events (AEs) were coded using the Medical Dictionary for Regulatory Activities, version 24.0.

## Discussion

During our study period, the Omicron BF.7 sublineage caused several outbreaks in Beijing. Our preliminary results in healthy adult workers within 72 h of contact with SARS-CoV-2-infected individuals showed that SA58 Nasal Spray was highly effective in preventing symptomatic COVID-19 and SARS-CoV-2 infection caused by Omicron BF.7 sublineage, which variants have shown significant escape of immunity in previous studies [19]. SA58 was able to significantly lower the risk of laboratory-confirmed COVID-19 by 80.82% (95%CI 52.41%−92.27%) and of SARS-CoV-2 infection by 61.83% (95%CI 37.50%−76.69%) in our study participants, which has far-reaching implications. This newly developed mAb may provide a new powerful countermeasure to tackle this cunning virus, which is currently circulating in China as a result of reopening of the country. Moreover, our study demonstrated that SA58 had a favourable safety profile and was well tolerated by healthy adults, with mild and short-lived symptoms of nasal dryness, runny nose, and nasal congestions observed among study participants. The intranasal administration of SA58 is novel and has some advantages over intramuscular injections of mAbs previously licensed and discontinued [11,12], as it is less invasive and more acceptable to recipients. Auto-administration with easiness of use may allow early administration, probably a key feature for prevention.

The median incubation period of SARS-CoV-2 was estimated to be five days, with most cases developing symptoms within 11.5 days of infection [20]. The distribution of the incubation period has implications for the use of SA58 as a PEP treatment, however separate analysis by duration of follow-up (<5 days vs. ≥5 days) did not show a difference in efficacy of SA58 in this study. SA58 contains mAbs that can potently neutralize a wider range of circulating Omicron variants in vitro, including BA.1, BA.2, BA.2.12.1, BA.3, BA.4/BA.5, BF.7, and other variants that have been tested so far [13]. The mAbs are not absorbed into blood and act as a physical barrier to stop the attachment and entry of viral particles into target cells of the throat and nasopharyngeal mucosa. The short half-life of SA58 in nasal mucosa suggests that the effect of SA58 is transient after administration and should be administered as early as possible to cover the incubation of SARS-CoV-2 infection. Since the incubation period of the Omicron variant was reported to be as short as three days [21], our selection of study participants within 72 h of contact with SARS-CoV-2-infected individual is deemed appropriate. Yet, the duration of case monitoring is less than three days for most study participants in this study. To evaluate the efficacy of SA58, a longer monitoring period of ≥11 days might be justified in future investigations.

There were 99 SARS-CoV-2 RT–PCR positive participants at baseline (*n* = 54) and within 24 h of the first drug administration (*n* = 45), suggesting that they have been infected at enrollment. These 99 participants were offered SA58 (*n* = 77) and placebo (*n* = 22). Our separate analysis by including these 45 participants who tested positive within 24 h of administration showed a significant drop of protective efficacy of SA58, from 80.82% to 50.30% for Symptomatic COVID-19, and from 61.83% to 32.69% for SARS-CoV-2 infection. It suggested that PEP with SA58 was unable or impotent to avert the disease course when SARS-CoV-2 infection had been firmly established in the body, highlighting the importance of early use of SA58 for the exposed again. On the other hand, one purpose of PEP with mAb is to slow the illness progression prior to the illness onset. Since the sample size was low in the current analysis, we did not evaluate the therapeutic effect of SA58 in lowering severity of symptoms or in shortening illness duration in these study participants. To explore the potential benefits of SA58 in slowing illness progression or in shortening its duration, we recommend evaluating this in upcoming studies.

Our study has several limitations. First, our study participants were confined to healthy workers generally young and healthy, which limits the generalizability of our study results to other populations, namely the elderly living in long-term care facilities, healthcare personnel who have frequent contacts with patients at increased risk of severe outcomes, and people who have underlying medical conditions. These populations have a higher risk of severe disease or death following SARS-CoV-2 infection and are more likely to benefit from PEP with mAbs. In the future, studies need to be conducted in these high-risk populations to expand the target population for SA58. Second, concurrent practices may impact on the observed effect of SA58. For example, in an attempt to block transmission and to quickly stop the potential outbreak, our infected study participants were managed at isolation facilities for highly infectious diseases, which lowers the risk of infection and may distort associations between treatment and disease outcome. Further assessment of SA58 in participants continuously exposed to SARS-CoV-2 in real-world situation is needed. Third, our study ended prematurely on December 9, 2022, two days after the Chinese government lifted its COVID-19 containment policy on December 7, 2022 [16], causing our follow-up of study participants to be insufficient. Short duration of follow-up might have had an impact on our study by detaining us to acquire a meaningful efficacy result. A lower proportion of symptomatic COVID-19 was observed in participants who had a follow-up duration of <3 days after the first positive RT–PCR test (6/35), as compared to those who had a follow-up ≥3 days (15/29), *p* = 0.007. Yet, the separate analyses by duration of follow-up showed similar results for efficacy estimates of SA58, highlighting the robustness of the study results and offering some kind of relief to this problem. Finally, in this preliminary analysis the sample size was low. The continuous follow-up of study participants and full-powered number of participants (e.g. *n* = 2300) is advised to confirm the efficacy and safety of SA58. To increase clarity and transparency of reporting, we recalculated the power of the study for observing a meaningful safety or efficacy endpoint. For adverse events that occur with a frequency of 0.5% or over, the probability of observing at least one event using the current sample size of participants who received SA58 (*n* = 901) in our study is approximately 0.99. For a meaningful efficacy endpoint, the sample size of the study would have a power of 0.8 to detect a significant RR of 0.3 and less between the treatment and placebo group.

## Conclusions

SA58 in healthy adults with early exposure to SARS-CoV-2 within 72 h has shown satisfactory efficacy and safety in reducing symptomatic COVID-19 cases and SARS-CoV-2 infections. SA58 as a potential PEP treatment in preventing COVID-19 should be further evaluated in high-risk populations who are at risk of severe outcomes following infection, e.g. the elderly, healthcare personnel and people with predisposing underlying illnesses.

## Supplementary Material

Supplemental Material

## Data sharing

De-identified individual participant-level data will be available upon written request to the corresponding author following publication.

## References

[1] Ikematsu H, Hayden FG, Kawaguchi K, et al. Baloxavir marboxil for prophylaxis against influenza in household contacts. N Engl J Med. 2020;383(4):309–320. 32640124 10.1056/NEJMoa1915341

[2] WHO Expert Consultation on Rabies: third report. [Internet]. World Health Organization. 2018. Available from: https://apps.who.int/iris/bitstream/handle/10665/272364/9789241210218-eng.pdf.

[3] Kuhar DT, Henderson DK, Struble KA, et al. Updated U.S. Public Health Service guidelines for the management of occupational exposures to HIV and recommendations for postexposure prophylaxis [Pamphlet (or booklet)]. 25/2013 Update. 2018. 10.1086/672271 23917901

[4] Redeker AG, Mosley JW, Gocke DJ, et al. Hepatitis B immune globulin as a prophylactic measure for spouses exposed to acute type B hepatitis. N Engl J Med. 1975;293(21):1055–1059. 1101065 10.1056/NEJM197511202932101

[5] Brunell PA, Ross A, Miller LH, et al. Prevention of varicella by zoster immune globulin. N Engl J Med. 1969;280(22):1191–1194. 4181206 10.1056/NEJM196905292802201

[6] Slifka MK, Amanna IJ, et al. Passive immunization. In: Orenstein W, Offit PA, Edwards KM, editors. Plotkin’s vaccines. Philadelphia, PA, US: Elsevier Health Sciences; 2018. p. 84–95.

[7] Zhu N, Zhang D, Wang W, et al. A novel coronavirus from patients with pneumonia in China, 2019. N Engl J Med 2020;382(8):727–733. 31978945 10.1056/NEJMoa2001017 PMC7092803

[8] Walker PGT, Whittaker C, Watson OJ, et al. The impact of COVID-19 and strategies for mitigation and suppression in low- and middle-income countries. Science. 2020;369(6502):413–422. 32532802 10.1126/science.abc0035 PMC7292504

[9] World Health Organization. WHO Coronavirus (COVID-19) Dashboard 2022. Available from: https://covid19.who.int/

[10] Strohl WR, Ku Z, An Z, et al. Passive immunotherapy against SARS-CoV-2: from plasma-based therapy to single potent antibodies in the race to stay ahead of the variants. BioDrugs. 2022;36(3):231–323. 35476216 10.1007/s40259-022-00529-7 PMC9043892

[11] U.S. Food and Drug Administration. Coronavirus (COVID-19) Drugs: U.S. Food and Drug Administration; 2023 [updated March 10; cited 2023 March 15]. Available from: https://www.fda.gov/drugs/emergency-preparedness-drugs/coronavirus-covid-19-drugs

[12] U.S. Food and Drug Administration. Emergency use authorizations for drugs and Non-vaccine biological products: U.S. Food and Drug Administration; 2023 [updated March 10; cited 2023 March 15]. Available from: https://www.fda.gov/drugs/emergency-preparedness-drugs/emergency-use-authorizations-drugs-and-non-vaccine-biological-products

[13] Cao Y, Jian F, Zhang Z, et al. Rational identification of potent and broad sarbecovirus-neutralizing antibody cocktails from SARS convalescents. Cell Rep. 2022 Dec;1:111845. 10.1016/j.celrep.2022.111845 PMC9712074 36493787

[14] Cao Y, Jian F, Wang J, et al. Imprinted SARS-CoV-2 humoral immunity induces convergent omicron RBD evolution. Nature. 2023 Feb;614(7948):521–529. 36535326 10.1038/s41586-022-05644-7 PMC9931576

[15] Si S, Jin C, Li J, et al. Safety and effectiveness of SA58 nasal spray against COVID-19 infection in medical personnel: an open-label, blank-controlled study – hohhot city, Inner Mongolia autonomous region, China, 2022. China CDC Weekly. 2023;5(10):218–222. 37006441 10.46234/ccdcw2023.040 PMC10061814

[16] The State Council PRC. ‘New Tens’ for the control and prevention of COVID-19. 2022 Dec 7. The State Council, P.R. China, 2022. Available from: http://www.nhc.gov.cn/xcs/gzzcwj/202212/8278e7a7aee34e5bb378f0e0fc94e0f0.shtml.

[17] National Health commission of the people’s republic of China. Protocol for prevention and control of COVID-19. Beijing: National Health Commission of the People’s Republic of China; 2022; Available from: http://www.nhc.gov.cn/yzygj/s7653p/202203/b74ade1ba4494583805a3d2e40093d88.shtml. 10.46234/ccdcw2020.082 PMC8392946 34594648

[18] NIH National Cancer Institute. Common terminology criteria for adverse events (CTCAE) version 5.0. 2017. Available from: https://evs.nci.nih.gov/ftp1/CTCAE/CTCAE_5.0.

[19] Cao Y, Wang J, Jian F, et al. Omicron escapes the majority of existing SARS-CoV-2 neutralizing antibodies. Nature. 2022;602(7898):657–663. 35016194 10.1038/s41586-021-04385-3 PMC8866119

[20] Lauer SA, Grantz KH, Bi Q, et al. The incubation period of coronavirus disease 2019 (COVID-19) from publicly reported confirmed cases: estimation and application. Ann Intern Med. 2020;172(9):577–582. 32150748 10.7326/M20-0504 PMC7081172

[21] Xin H, Wang Z, Feng S, et al. Transmission dynamics of SARS-CoV-2 omicron variant infections in Hangzhou, Zhejiang, China, January-February 2022. Int J Inf Dis. 2022;126:132–135. 36511336 10.1016/j.ijid.2022.10.033 PMC9616478

